# Mutations in the *TaPIN1* peptidyl prolyl isomerase gene in *Theileria annulata* parasites isolated in Sudan

**DOI:** 10.1016/j.ijpddr.2019.11.001

**Published:** 2019-11-26

**Authors:** Bashir Salim, Elisha Chatanga, Guillaume Jannot, Ehab Mossaad, Ryo Nakao, Jonathan B. Weitzman

**Affiliations:** a Department of Parasitology, Faculty of Veterinary Medicine, University of Khartoum, P.O. Box 32, Khartoum North, Sudan; b Laboratory of Parasitology, Graduate School of Infectious Diseases, Faculty of Veterinary Medicine, Hokkaido University, N18 W9, Sapporo, Hokkaido, 060-0818, Japan; c Université de Paris, Epigenetics and Cell Fate, CNRS, F-75013, Paris, France; d Department of Pathology, Parasitology and Microbiology, College of Veterinary Medicine, Sudan University of Science and Technology, P.O. Box 204, Khartoum, Sudan

**Keywords:** Drug-resistance, Prolyl isomerization, Point mutations, Markers, *Theileria*, *Theileriosis*

## Abstract

The tick-borne parasite *Theileria annulata* is the causative agent of tropical theileriosis or Mediterranean theileriosis. Infection of bovine leukocytes by the obligate intracellular parasites induces proliferative and invasive phenotypes associated with activated signaling pathways. The transformed phenotypes of infected cells are reversible by treatment with the theilericidal drug buparvaquone. Recent reports of resistance to buparvaquone in Africa and Asia highlight the need to investigate the mechanisms and prevalence of drug resistance. We screened 67 *T. annulata* isolates from Sudan to investigate mutations in the *T. annulata* prolyl isomerase I gene (*TaPIN1*). The secreted TaPin1 interacts with host proteins to induce pathways driving oncogenic transformation and metabolic reprogramming. We found an Alanine-to-Proline mutation at position 53 (A53P) in the catalytic loop that was previously found in Tunisian drug-resistant samples. This is the first study reporting independent confirmation of the A53P mutation in geographically isolated samples. We found several additional mutations in the predicted N-terminal signal peptide that might affect TaPin1 processing or targeting. We found that many parasites also share mutations in both the *TaPIN1* and the *cytochrome b* genes, suggesting that these two genes represent important biomarkers to follow the spread of resistance in Africa, the Middle East and Asia.

## Graphical abstract

**Figure:**
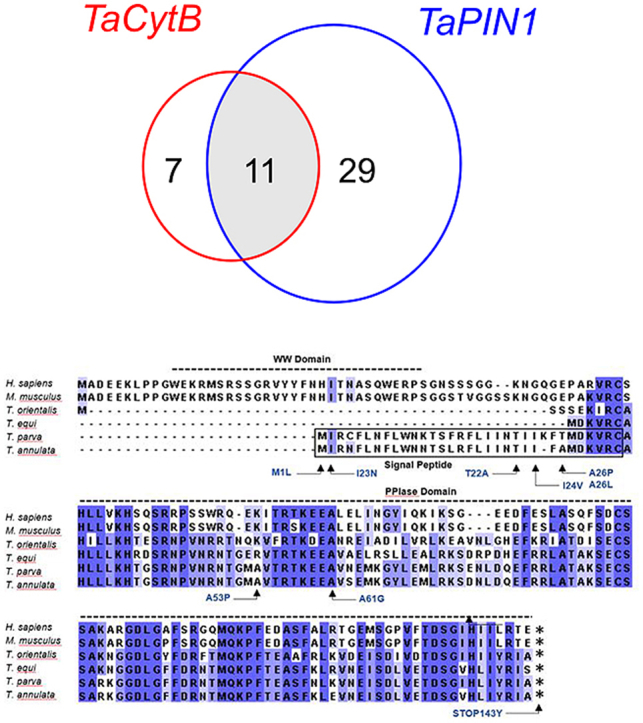

## 1 Introduction

Bovine Tropical Theileriosis (BTT) is a lymphoproliferative, tick-borne disease of cattle caused by the protozoan parasite *Theileria annulata.* The intracellular *Theileria* parasites transform mammalian host cells by inducing cancer-like phenotypes, such as immortalization, dissemination and hyper-proliferation of the infected leukocytes (Morrison, 2009). During the pathogenesis of the disease, the infected host cells spread throughout the animal, causing enlargement of lymph nodes and eventual lethality. Theileriosis has been reported in southern Europe, North Africa, India, China and across Asia with substantial socio-economic impact on farming and veterinary health.

The molecular mechanisms underlying host cell transformation by *Theileria* parasites likely involve changes in signaling pathways that affect the host epigenome and cause changes in host cell gene/protein expression and activation (Cheeseman and Weitzman, 2015; Lüder et al., 2009; Morrison, 2009). For example, *Theileria* infection of bovine leukocytes modulates oncogenic signaling pathways such as JNK and IKK (Heussler et al., 2002; Lizundia et al., 2007) and activation of host transcription factors such as c-Myc, AP-1 and HIF-1α (Chaussepied et al., 1998; Dessauge et al., 2005; Marsolier et al., 2013; Medjkane et al., 2014; Metheni et al., 2015). Furthermore, *Theileria* parasites interact with host microtubules and recruit end-binding protein 1 to the parasite surface (von Schubert et al., 2010; Woods et al., 2013).

We recently identified the parasite-encoded Peptidyl Prolyl Isomerase Pin1 (designated TaPin1) in *T. annulata* as a secreted protein (Marsolier et al., 2015). The parasite TaPin1 protein can interact with host cell proteins and modulates oncogenic signaling pathways For example, the TaPin1 prolyl isomerase interacts with the host ubiquitin ligase Fbw7, leading to its degradation and subsequent stabilization of c-Jun which promotes host cell transformation (Marsolier et al., 2015). TaPin1 can also stabilize the host pyruvate kinase isoform M2 (PKM2) leading to HIF-1α-dependent metabolic reprogramming (Marsolier et al., 2019). Parasite-induced transformation of host cells is reversible with the anti-*Theilerial* drug buparvaquone (McHardy et al., 1985). Buparvaquone administration results in significant damage of both schizont and piroplasm stages of *T. annulata* causing reduction of these parasitic stages to low or undetectable levels within 7 days (Mhadhbi et al., 2010). At least one mechanism of buparvaquone action may be via inhibition of TaPin1 isomerase activity (Marsolier et al., 2019, 2015).

The socio-economic impact of Theileriosis has been exacerbated by the emergency of resistance to buparvaquone, the frontline drug in the treatment of BTT. Recently, cases of buparvaquone-resistant *T. annulata* isolates were reported in Sudan, Tunisia, Iran and Turkey (Chatanga et al., 2019; Mhadhbi et al., 2015, 2010; Sharifiyazdi et al., 2012). Drug-resistance poses a serious threat to the dairy industry in the endemic countries where the disease continues to cause significant losses due to deaths, as well as operational costs to mitigate the impact of the disease. Several of these cases have been linked to mutations in the parasite mitochondrion cytochrome B gene (Chatanga et al., 2019; Mhadhbi et al., 2015; Sharifiyazdi et al., 2012). We also reported mutations in the *TaPIN1* gene in buparvaquone-resistant parasite isolated in Tunisia (Marsolier et al., 2015). Specifically, an alanine to proline mutation at residue 53 (A53P) in the catalytic loop of TaPin1 caused loss of sensitivity to buparvaquone in recombinant TaPin1 protein (Marsolier et al., 2015). Both *in vitro* and *in vivo* experiments demonstrated that mutant TaPIN1 was not inhibited by PIN1 inhibitors, such as anti-*Theileria*l drug buparvaquone (Marsolier et al., 2015).

Here, we report studies on *T. annulata* resistance to buparvaquone in isolates from Sudan. We sequenced the parasite *TaPIN1* gene to determine the extent of mutations in the resistant isolates. Our results show that mutations in *TaPIN1* may be widespread in drug-resistant parasites.

## 2 Materials and methods

### 2.1 Ethical considerations

We examined sixty-seven DNA samples from blood from cattle clinically diagnosed, and confirmed by both Giemsa blood smears and polymerase chain reaction (PCR), to be infected with *T. annulata* in Sudan. This was a parallel study of our initial study (Chatanga et al., 2019) which was approved by the University of Khartoum, Sudan (Approval no. 2017BS), and Sudan University of Science and Technology, Khartoum, Sudan (Approval no.28–46), and informed consent was sought from animal owners. Thus, no separate approval was required.

### 2.2 Geographical study area

The samples were collected from Omdurman (15.38 °N, 32.28 °E) (n = 17) and Khartoum North (15.63 °N, 32.56 °E) (n = 31) and Khartoum (15.55 °N, 32.53 °E) (n = 19) in December 2016 to March 2018. These samples were collected from farms where buparvaquone is actively used in the treatment of bovine tropical theileriosis in Khartoum state, Sudan. These samples were collected from cattle clinically diagnosed and confirmed by Giemsa blood smears to be infected with *T. annulata*. These samples came from farm owners who specifically reported that these animal were not responding to buparvaquone treatment at the normal dose.

### 2.3 DNA extraction and PCR conditions

DNA was extracted using DNAzol BD Reagent (Invitrogen, MA, USA) or QIAmp DNA Blood Mini kit protocol (Qiagen, Tokyo, Japan) and stored at −20 °C until analysis. PCR was performed using the primers *TaPIN1* forward 5′-GTCTGTCAAATAGGTAGAAATC-3′ and reverse 5′-GAGAGGAAGTTGAATCAAACAT-3′ to amplify the 526 bp encompassing the full length of the gene. The reaction was conducted in a 25 μl reaction mixture containing 12.5 μl of 2xGflex PCR Buffer (Mg^2+^, dNTP plus), 0.5 μl of Tks Gflex DNA Polymerase 1.25 units/μl (TaKaRa Bio Inc., Shiga, Japan), 200 nM of each primer, 1.0 μl of template DNA and molecular-grade water. The cycling conditions were initial denaturation at 94 °C for 1 min followed by 35 cycles of denaturation at 98 °C for 10 s, annealing at 57 °C for 15 s, extension at 68 °C for 45 s and final extension at 68 °C for 5 min. The amplicons were electrophoresed in 1.5% agarose gel stained with Gel-Red (Biotium, Hayward, CA, USA) and visualized under UV light.

### 2.4 Sequence processing and analysis

The amplicons were purified using the NucleoSpin Gel and PCR Clean Up Kit (TaKaRa Bio Inc.) and sequenced using the Big Dye Terminator version 3.1 Cycle Sequencing Kit (Applied Biosystems, Foster City, CA, USA) utilizing an ABI Prism 3130 x genetic analyser (Applied Biosystems) according to the manufacturers' instructions. Two sequence fragments were generated for each sample and ATGC software version 9.1 (GENETYX Corporation, Tokyo, Japan) was used for manual editing to correct possible base calling errors and remove primer annealing sites. The regions of unambiguously aligned sequences were eventually joined to reconstruct sequences of 482bp of *T. annulata TaPIN1* gene which were retained for final analysis.

### 2.5 Sequence analysis

The 482 bp nucleotide sequences corresponding to the PPlase TaPIN1 were successfully sequenced for 67 *T. annulata* samples from Sudan and the sequences obtained in the present study were submitted to the Mendeley Data Repository. These consensus sequences were translated into 143 amino acids and the protein multiple alignment was generated by Molecular Evolutionary Genetics Analysis (MEGA) and viewed with Jalview softwares respectively (Waterhouse et al., 2009) using TaPIN1 (PiroplasmaDB: TA18945) as reference sequence.

## 3 Results

### 3.1 Analysis of the *TaPIN1* sequences in Sudanese isolates

**Fig. 1:**
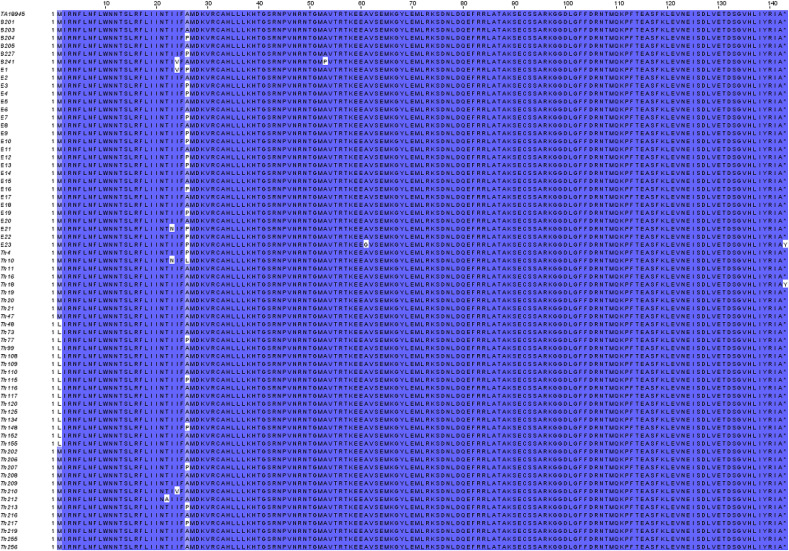
Multiple sequence alignment of TaPin1 *T. annulata* proteins from Sudanese isolates. Amino acids that differ from the reference TaPin1 sequence are shown in white.

**Fig. 2:**
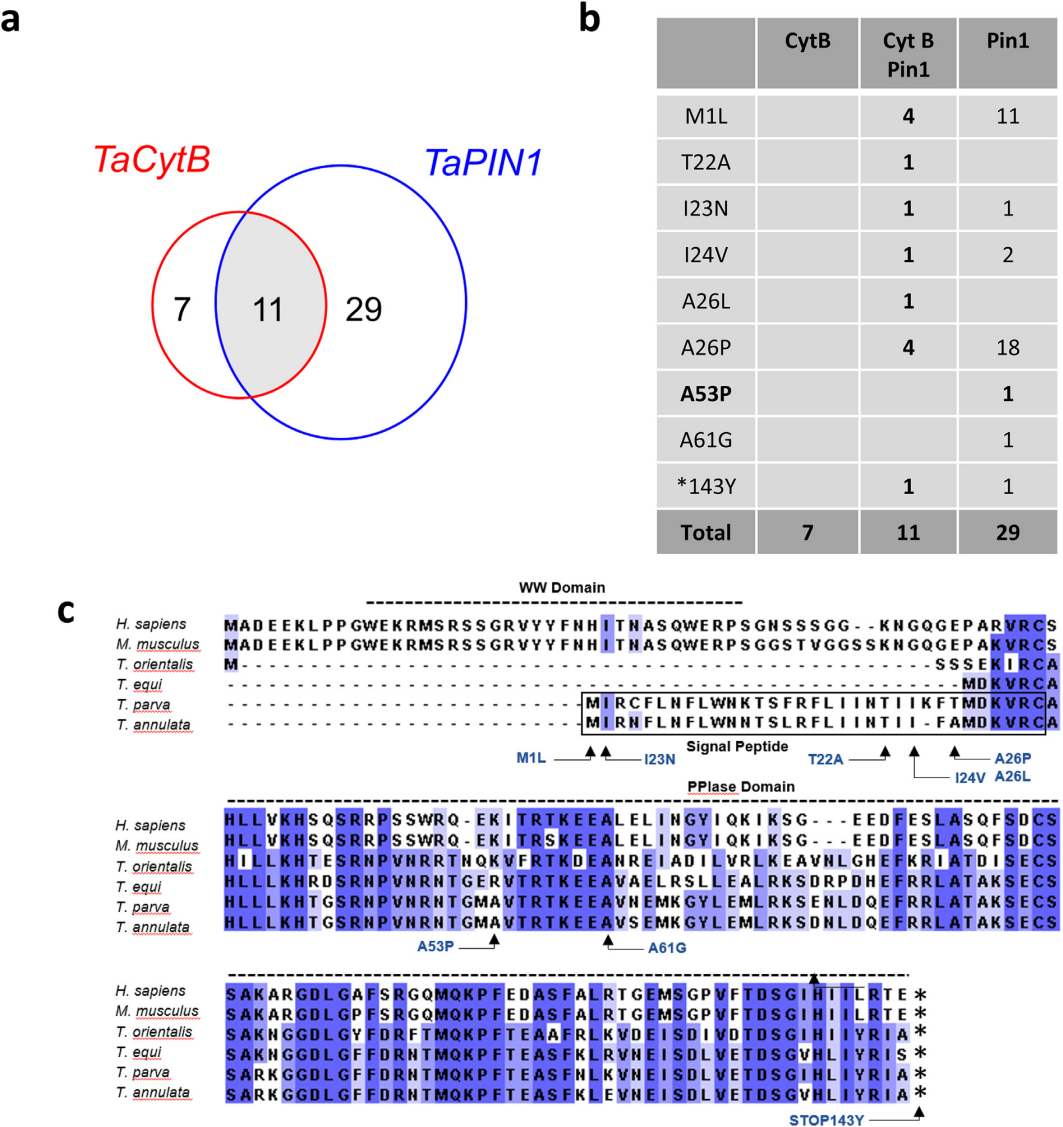
Comparison of mutations in *T. annulata Cytochrome b* and *TaPIN1* genes. a. Schematic representation of the number of isolates with mutation in the Cyt b or Pin1 gene and the number of isolates with mutations in both genes. b. Details of the number of isolates identified with mutations at each position. The number of isolates with only TaPIN1 mutations (right) or with mutations in both genes (center). c. Sequence alignment of Pin1 proteins in *H. sapiens*, *M. musculus*, *T. orientalis*, *T. equi*, *T. parva* or *T. annulata* showing the predicted signal peptide (boxed) in TaPin1 and TpPin1 and the positions of each mutation identified in this study.

We aligned the amino acid sequences corresponding to the TaPin1 protein from the 67 Sudanese isolates and compared with the annotated, reference sequence for *T. annulata* PIN1 PPlase (TA18945). We discovered 8 positions in the isolates from Sudan where the sequences differed from the reference TA18945 sequences (Fig. 1, Table 1). Interestingly, nearly two thirds of the isolate sequences (40/67) had a sequence that differed from the annotated TaPin1 sequence. This is even higher than the number of mutations detected (18 sequences) in the *T. annulata* cytochrome b gene in our previous study (Chatanga et al., 2019). And nine of the isolates presented two mutations in the *TaPIN1* gene (Fig. 1, Table 1). Sixteen of the isolate sequences had a M1L mutation and two of the sequences had a STOP143Y mutation. These mutations would likely result in the loss of TaPin1 protein, so we cannot speculate on their functional relevance. A comparison of the *TaPIN1* results with analysis of the *cytochrome b* mutations revealed that 11 (16%) of the isolates presented mutations in both the parasite genes (Fig. 2).

**Table 1:** Summary of mutations in *TaPIN*1 sequences of *T. annulata* isolates from Sudan. At each amino acid position, the TA18945 reference sequence is shown together with the mutation detected in the Sudanese isolates (left column) and the number of sequences affected (brackets).

Amino acid position	1	22	23	24	26	53	61	143
TaPIN1 TA18945	M	T	I	I	A	A	A	STOP
Th48,Th73,Th77,Th99,Th108,Th109,Th110,Th115,Th116,Th117,Th120,Th125,Th134,Th148,Th152,Th155	L (16)							
Th212		A (1)						
Th10,E21			N (2)					
B241,E1,Th210				V (3)				
Th10					L (1)			
B204,B227,E1,E3,E4,E7,E9,E10,E12,E13,E16,E19,E21,E22,E23,Th4,Th77,Th115,Th148,Th207,Th213,Th217					P (22)			
B241						P (1)		
E23,							G (1)	
E23,Th18								Y (2)

### 3.2 Mutations in the *TaPin1* sequences in samples from Sudan

In a previous study of buparvaquone-resistant parasites isolated in Tunisia, we reported the identification of a mutation (A53P) in the catalytic loop of TaPin1 which could account for drug resistance (Marsolier et al., 2015). TaPin1 protein carrying this mutation maintained prolyl isomerase activity, measured by an *in vitro* assay for catalytic isomerization, but was no longer inhibited by buparvaquone treatment (Marsolier et al., 2015). Interestingly, one of the Sudanese samples (isolate B241) contained the same A53P mutation that predicts a functional isomerase which is not functionally inhibited by buparvaquone (Fig. 1, Table 1). This is the first independent confirmation of this mutation in two geographically distinct isolates.

Our initial analysis of the Tunisian isolates had identified additional mutations that we did not pursue at the time as they fell within the sequence that we predicted as a signal peptide that could be cleaved upon TaPin1 secretion (Marsolier et al., 2015). These included, T22I and A26L. Intriguingly, we found a number of mutations in the Sudanese isolates that also affected residues in the proposed signal peptide. These included T22A, I23N, I24V, A26L and A26P (Fig. 2, Table 1). Thus, three of the positions mutated in the Tunisian samples (residues 22, 26 and 53) are also mutated in the Sudanese samples (accounting for 25 of the isolated samples).

## 4 Discussion

The emergence of resistance to anti-parasite drugs should drive our search for drug targets and the mechanisms of resistance. BTT is a case in point, as resistance to buparvaquone, the leading drug treatment for Theileriosis, has been observed across North Africa. Here, we report the discovery of mutations in the *TaPIN1* gene in isolates from Sudan. Indeed, 40 of the 67 Sudanese sequences (60%) have a mutation that differs from the *TaPIN1* reference sequence. Notably, we report the first independent confirmation of the A53P mutation previously characterized in drug-resistant *T. annulata* parasites from Tunisia (Marsolier et al., 2015). This result strengthens our initial claim that TaPin1 could be a direct target for buparvaquone as the same functional mutation has now been described in two isolates from distant geographical locations. The mutation to Proline in the TaPin1 catalytic loop is likely to induce the conformational alterations affecting access of buparvaquone to the PPlase active site.

We also observed several other mutations, particularly at positions 22, 23, 24 and 26. Interestingly, two of these (T22I and A26L) were also found in the Tunisian samples (Marsolier et al., 2015). These positions are unlikely to affect the catalytic activity directly, but they could affect the secretion, processing or targeting of the TaPin1 protein. As noted previously, the signal peptide sequence is not found in non-transforming *Theileria* species, but is conserved in *T. parva*. We do not currently have any experimental evidence that the predicted signal peptide is actually cleaved. It is possible that this N-terminal sequence is critical for targeting to a particular organelle or for proteins interactions, and this merits future investigation. In addition, two sample sequences had a *143Y mutation, representing a change in the STOP codon; it is unclear at the moment what the functional consequences of a longer protein could be.

Another important finding of our study is the overlap with mutations in the *T. annulata cyt*ochrome b gene. Several reports have shown mutations in this gene in drug-resistant isolates from around the world (Chatanga et al., 2019; Mhadhbi et al., 2015, 2010). But this is the first time that both genes have been investigated in the same samples and the high degree of overlap suggests that perhaps both genes contribute together to drug resistance. Futhermore, while 40 out of the 67 samples have a mutation in either of these two genes, we currently have no indication of what could account for the bupavaquone-resistance in the other 27 samples, suggesting that additional genes remain to be discovered. Our results highlight the need to develop molecular marker(s) and phenotypic tests to detect the occurrence of resistance and determine its prevalence in the population (Mhadhbi et al., 2015). *TaPIN1* and *cytochrome b* should both be used as markers to screen resistant strains of *T. annulata* to buparvaquone in field isolates.

In conclusion, this study of field isolates from Sudan extends our understanding of buparvaquone resistance in *T. annulata* parasites. We confirm the importance of the A53P mutation in the catalytic loop and identify additional N-terminal mutations that could affect TaPin1 processing or targeting. Finally, the high frequency of mutations in the *TaPIN1* and *cytochrome b* genes suggests that both genes should be used as molecular markers to detect the spread of resistance in the field across endemic areas in Africa and the Middle East.

## Data availability

All data generated or analyzed during this study is publicly available on the Mendeley Data Repository.

## Declaration of competing interest

All authors have no competing interests.
